# Nutritional Strategies to Prevent Lens Cataract: Current Status and Future Strategies

**DOI:** 10.3390/nu11051186

**Published:** 2019-05-27

**Authors:** Andrea J Braakhuis, Caitlin I Donaldson, Julie C Lim, Paul J Donaldson

**Affiliations:** 1 Discipline of Nutrition, Faculty of Medical and Health Sciences, the University of Auckland, Auckland 1142, New Zealand; a.braakhuis@auckland.ac.nz (A.J.B.); c-donaldson@hotmail.com (C.I.D.); 2 Department of Physiology, Faculty of Medical and Health Sciences, New Zealand National Eye Centre, the University of Auckland, Auckland 1142, New Zealand; j.lim@auckland.ac.nz

**Keywords:** dietary antioxidants, antioxidant supplements, lens, cataract

## Abstract

Oxidative stress and the subsequent oxidative damage to lens proteins is a known causative factor in the initiation and progression of cataract formation, the leading cause of blindness in the world today. Due to the role of oxidative damage in the etiology of cataract, antioxidants have been prompted as therapeutic options to delay and/or prevent disease progression. However, many exogenous antioxidant interventions have to date produced mixed results as anti-cataract therapies. The aim of this review is to critically evaluate the efficacy of a sample of dietary and topical antioxidant interventions in the light of our current understanding of lens structure and function. Situated in the eye behind the blood-eye barrier, the lens receives it nutrients and antioxidants from the aqueous and vitreous humors. Furthermore, being a relatively large avascular tissue the lens cannot rely of passive diffusion alone to deliver nutrients and antioxidants to the distinctly different metabolic regions of the lens. We instead propose that the lens utilizes a unique internal microcirculation system to actively deliver antioxidants to these different regions, and that selecting antioxidants that can utilize this system is the key to developing novel nutritional therapies to delay the onset and progression of lens cataract.

## 1. Introduction

Lens cataract is the leading cause of visual impairment and blindness worldwide [1,2]. It has been estimated that over 68% of people over 79 years of age have some form of reduced lens opacity or cataract [3], with disease incidence increasing with age. Clinically four main forms of lens cataract are recognised: sub-capsular, cortical, nuclear and mixed (nuclear and cortical). Of these classes, diabetic cortical cataract and age related nuclear (ARN) cataract are the most common. Population growth, sedentary lifestyles, unhealthy diets and an increasing prevalence of obesity are increasing the number of people with diabetes mellitus. Worldwide more than 285 million people are affected by diabetes mellitus with the number expected to increase to 439 million by 2030 [4]. A frequent complication of both type 1 and type 2 diabetes is diabetic cortical cataracts which occur 2–5 times more frequently in patients with diabetes, and occur at an earlier age [5]. In parallel, our aging population is growing at a remarkable rate with the population aged 60 years or over predicted to double to 2.1 billion by 2050 [6]. Age is major risk factor for cataracts with the current estimate of cataracts to afflict more than 20 million people worldwide. Given our globally aging population, the social and economic costs of lens cataract are quite staggering and the demand for cataract surgery far exceeds limited public health resources. In the USA alone, cataract surgery is the most commonly performed surgical procedure and costs around $3.5 billion per year. In Australia, where it has been estimated that the population will increase by 22% between 1996 and 2021, the incidence of age-related cataract will disproportionately increase by 76% during the same period [7]. While in New Zealand, it is predicted that by 2020 almost 22,800 New Zealanders will have vision loss and about 2,000 will be blind, due to cataract [8]. Annually some 16,000 cataract surgeries are performed in New Zealand, and in 2009–2010 more than 30 million dollars was spent on public inpatient and day stay services for cataract operations [8]. 

Thus, it is expected that the burden of cataract-impaired vision will increasingly outstrip the resources available for its surgical treatment. The alternative approach is to delay the onset of cataract. It has been predicted that delaying the onset of cataract by 10 years will halve the incidence of ARN cataract, greatly reducing the need for, and expense associated with, surgical intervention [9]. Since cataract is a progressive disease of old age, any medical therapy devised would need to be taken by a large cohort of the population over many years to be effective. Hence, it is likely that the most effective treatment will be in the form of nutritional supplements, either delivered orally or via eye drops. However, while there is evidence that diet can influence the onset and incidence of cataract (see Table 1), the results from trials into the use of nutritional supplements to prevent cataract have had mixed results (see Table 2). For example, the protective effects of antioxidant Vitamin supplements have been advocated in a number of studies [10,11,12], but a randomized, placebo-controlled trial of Vitamin E and cataract [13], and the Age-Related Eye Disease Study involving use of a high-dose formulation of Vitamin C, Vitamin E, and beta carotene [14], revealed no significant benefits of antioxidants. Furthermore, in large, prospective, population-based cohorts it was found that high-dose Vitamin C or Vitamin E supplements had a statistically significant increased risk of age-related cataract [15], most likely due to the pro-oxidative properties of Vitamins C and E at high doses reported in *in vitro* studies [16,17]. While in a large-scale randomized trial of women at high risk of cardiovascular disease, daily supplementation with a combination of folic acid, vitamin B_6_, and vitamin B_12_ had no significant effect on cataract, but may have increased the risk of cataract extraction [18]. However, high intake of dietary vitamin K_1_ was associated with a reduced risk of cataracts in an elderly Mediterranean population even after adjusting by other potential confounders [19]. These inconsistencies are in part firstly because lens cataract manifests as multiple disease phenotypes and different nutritional supplements may be more effective on a specific type of cataract, and secondly from a general lack of knowledge on how nutrients are delivered to, and metabolized in the distinctly different regions of the lens. Confounding this variability are differences in the methodology of the studies used to assess the efficacy of a dietary intervention on reducing the incidence of cataract. 

In this review, we first characterize the etiology of the main types of lens cataract, and then provide a summary of the evidence for and against the efficacy of nutritional status to delay or prevent the onset of these different sub-types of lens cataract. Then we provide an overview of lens structure and function to highlight how an increased understanding of these properties will be required to design more targeted nutritional strategies to reduce the incidence of cataract. 

## 2. The Etiology of Lens Cataract

Loss of lens transparency manifests itself clinically as cataract, the leading cause of blindness worldwide [2,20]. The two most common types of age-related cataract, cortical and nuclear cataract, have different damage phenotypes and are associated with a loss of transparency in the lens cortex and nucleus, respectively [21].

### 2.1. Cortical Cataract

**Figure 1:**
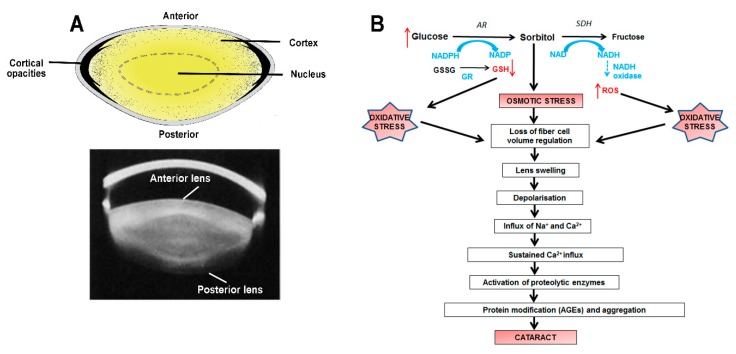
Cortical cataracts. (**A**) Location of the cortical cataract subtype. *Top panel:* diagram showing the opacities that form in the lens cortex. *Lower panel:* Scheimpflug slit-lamp photographic image revealing a cortical cataract. (**B**) Molecular mechanisms involved in the pathogenesis of diabetic cortical cataract. An increase in glucose leads to a decrease in GSH and an increase in reactive oxygen species (ROS) as indicated by the red arrows. The induced osmotic and oxidative stress work synergistically to inhibit the ability of fibre cells to regulate their volume. This leads to cell swelling, depolarization and an influx of sodium and calcium ions. The accumulation of calcium ions results in the activation of calcium-dependent proteases, which target cytoskeletal and crystallin proteins. Furthermore, proteins are modified by the formation of advanced glycation end (AGEs) products, which are known to alter the structure and function of crystallins, resulting in an increase in insoluble proteins, the formation of high molecular weight aggregates, and cataract.

Clinically, cortical cataract presents as wedge shaped or radial spoke opacifications in the lens cortex (Figure 1A), and is particularly prevalent in the elderly or diabetic patients [22]. Cortical cataracts tend to be associated with significant astigmatic shifts [23], caused by asymmetrical refractive index changes within the lens cortex [24]. This change in refractive index is most likely due to accumulation of fluid in the lens cortex since, at the cellular level, this light scattering is due to a discrete localized zone of tissue liquefaction surrounded by cells that have a normal morphological structure [25].

Animal models such as the streptozotocin (STZ) rat, a model used to chemically induce Type 1 diabetes by destroying pancreatic betacells, and galactose fed animals, a model for obese Type 2 diabetes, have been used to understand the mechanisms of diabetic cataract formation. These models have been commonly used because diabetes or galactosemia can be induced rapidly and effectively resulting in formation of “fast” sugar lens cataract. From these animal studies, the consensus view was that high levels of the impermeable osmolyte, sorbitol, produced from excess glucose by the enzyme aldose reductase (AR), initiates osmotic stress, resulting in the attraction of fluid, lens fiber cell swelling and tissue liquefaction [26,27,28]. Based on this view, considerable attention was focused on the development and testing of AR inhibitors, which were proven to be very successful in ameliorating diabetic cataract in rats and dogs [29], but ineffective in humans. The failure of aldose reductase inhibitors to slow the progression of cataract in humans lies with the differences in AR activity and polyol accumulation between rats and humans, with humans exhibiting low levels of AR activity relative to rat. In addition, the acute animal models replicate the fast development of cataract that occurs in diabetic patients with uncontrolled hyperglycemia. However, most diabetic patients are able to control their blood glucose reasonably well and so such acute cataract development is rarely seen. Instead, the majority of adult diabetic patients typically develop cataract after having suffered from diabetes for several years. Therefore, while the initiating mechanism in the development of diabetic cataract is osmotic stress, it is now believed that oxidative stress generated by polyol pathway activity [30], impairs the ability of the lens over time to regulate its volume, resulting in slow developing cataract formation [31]. This suggests that osmotic and oxidative stress work synergistically to cause a loss of cell volume and tissue liquefaction in the lens cortex (Figure 1B).

### 2.2. Age-Related Nuclear (ARN) Cataract

**Figure 2:**
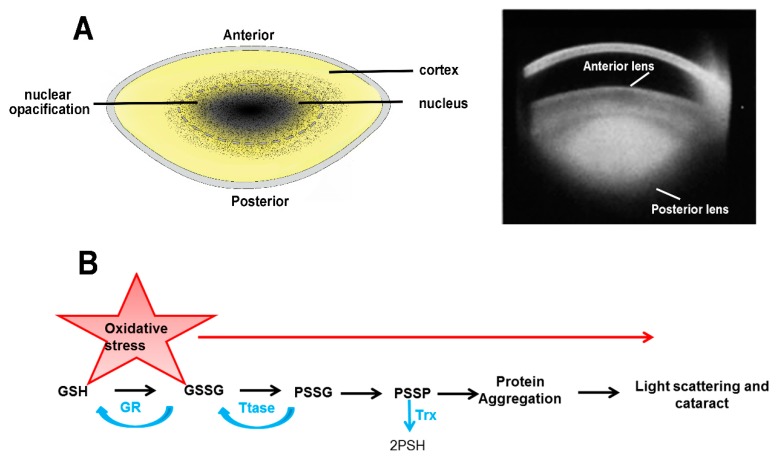
Nuclear cataracts. (**A**) Location of the nuclear cataract subtype. *Left panel:* diagram showing the opacities that form in the lens nucleus. *Right panel:* Scheimpflug slit-lamp photographic image revealing a nuclear cataract. (**B**) Molecular mechanisms involved in the pathogenesis of age-related nuclear cataract. GSH levels are maintained at high levels within the lens by a combination of pathways including regeneration of oxidised GSH (GSSG) back to GSH via the enzyme glutathione reductase (GR) as well as repair enzymes thioltransferase (Ttase), which dethiolate protein mixed disulfides, such as protein bound GSH (PSSG), and thioredoxin (TrX), that dethiolates protein-protein disulphides (PSSP). In age related nuclear cataract, depletion of GSH levels in the nucleus, but not the lens cortex, results in significant oxidation of nuclear proteins, an increase in protein mixed disulphides, formation of protein-protein disulfide bonds, protein aggregation, loss of protein solubility, increased yellowing of the lens nucleus, and eventual nuclear cataract formation.

Age-related nuclear cataract is initiated in the central core of the lens, which contains primary fiber cells that were initially laid down during embryonic development (Figure 2A). Clinically, age-related nuclear cataract appears as a browning or brunescence of the lens nucleus [32]. In contrast to cortical cataract, the morphology of the cells in the lens nucleus from nuclear cataract patients reveal no major structural distortions [33,34]. Instead, nuclear cataract is associated with the extensive loss of protein sulfhydryl groups, with over 90% of cysteine residues and ~50% of methionine residues being found oxidized in nuclear proteins in lenses obtained from patients with ARN cataract [35,36,37,38]. Accompanying the loss of protein sulfhydryl groups is an increase in protein-thiol mixed disulfides [39,40], and an increase in the water insoluble fraction [32,38], which culminate in the formation of protein-protein disulfides (PSSP), and other cross-linkages that lead to protein aggregation and light scattering. This series of biochemical changes has been extensively reviewed [41,42,43] and there is general agreement that oxidative stress is the major contributing factor to age-related nuclear cataract formation (Figure 2B). The unresolved question in nuclear cataract research now centers on how the normally robust oxygen radical scavenger systems present in the lens fail with advancing age, and initiate the observed protein aggregation specifically in the nucleus of the lens.

At younger ages, the human lens is normally protected against oxidative damage by a robust oxygen radical scavenger system, which utilizes GSH as its principal antioxidant to detoxify reactive oxygen species (ROS) [44]. A GSH concentration gradient exists in the lens, with GSH levels highest in the cortex and lowest in the nucleus. The high levels of GSH in the outer cortex are maintained by a combination of synthesis of GSH from its precursor amino acids; cysteine, glutamate and glycine [45]; direct uptake of GSH from the aqueous [46,47]; and the recycling of GSSG to GSH by glutathione reductase (GR), which utilizes NADPH, generated by the metabolism of glucose, as a reducing equivalent [44,48]. In contrast, cells in the lens nucleus have lost their capacity to synthesise GSH [49], and rely on anaerobic metabolism to produce their energy requirements [50]. Therefore, mature fiber cells can only maintain their GSH levels by delivery of GSH to the nucleus and/or local regeneration of GSH by GR. In ARN cataract, cortical fiber cells maintain their GSH levels, but GSH levels in the nucleus are significantly depleted (< 2mM) [51,52]. These observations have led to the hypothesis that ARN cataract is due to an inability to maintain GSH levels in the lens nucleus due to a failure to deliver GSH to the lens centre [53]. As a result, dietary supplementation with antioxidants has been extensively studied as a potential way to delay or prevent cataracts by countering the negative effects of oxidative stress. In this regard, GSH supplementation would appear to be the most obvious antioxidant to administer to restore the depleted levels of GSH observed in the nucleus of the old lens. However, GSH is poorly absorbed by an oral route mainly due to the action of an intestinal enzyme, the γ-glutamyl transpeptidase (GGT) which degrades GSH [54]. As a result, it’s low bioavailability would mean that very little GSH would reach the lens, let alone the lens nucleus, and so other strategies for enhancing antioxidant levels in the lens have been investigated. 

## 3. Evidence for and Against Nutritional Strategies to Prevent Lens Cataract

Although the pathogenesis of cortical and age related nuclear cataract are different, oxidative damage has been implicated as an underlying cause of the distinctly different damage phenotypes [30,31,42,43]. Because of this link between oxidative stress and cataract formation, it has been proposed that topical application and/or dietary interventions with antioxidants can be used as therapies to delay or prevent cataract progression. Hence, multiple studies have been conducted to assess the efficacy of such nutritional interventions. Unfortunately, a simplistic view of supplementing the lens with antioxidants has proven to be ineffective, and in some cases detrimental, in delaying cataract progression. This in part may be due to the emerging recognition that reactive oxygen species act as important modulators of redox signalling critical in maintaining normal metabolism and cellular processes. As such, antioxidant supplementation may in fact be counterproductive, eliminating physiological reactive oxygen species required for normal redox signaling and cellular function [55,56,57,58]. Therefore, in developing an antioxidant based cataract therapy, it is important to identify not only an appropriate antioxidant to minimise oxidative stress specifically in the lens, but also to consider how this antioxidant will be delivered at levels that will effectively restore antioxidant balance in the different lens regions to delay the formation of a specific type of cataract. 

### Effectiveness of Antioxidant Therapies on Cataract Progression—A Survey of the Literature

To gain a snap shot of the current effectiveness of antioxidant therapies to reduce the incidence of cataract formation, a survey of the literature was performed. This survey of the literature utilized Google Scholar to extract key references from texts and publications published in English from 2000 onwards, and utilized the search terms cataracts / vision AND antioxidants / supplements / nutrition. Research involving case-control, observational and randomised control design were included, but only if they investigated dietary antioxidants (including lutein, xanthine, Vitamin C, E, A, selenium, polyphenols) and/or fruit and vegetable intake on cataract incidence or progression. The initial search generated 15,900 publications, which once customized to publication date, was reduced to 14,500 articles. Of this search, the first 2900 articles were reviewed by title, representing up to page 30 of the database search. 2845 publications were excluded on the basis of the predefined criteria (predominantly a lack of relevancy, i.e. non-cataract), leaving 55 publications that were subjected to independent review in full by two authors (CD, AB). Disagreement between authors on their inclusion/exclusion was discussed, and a further 29 publications were excluded following full text review. In addition to the primary research articles, there were 14 reviews on the topic that proved useful for background evidence [1,3,59,60,61,62,63,64,65,66,67,68,69,70]. For ease of presentation, the results of this search are separated into the effects of whole dietary (Table 1), or topical supplement (Table 2) interventions on the incidence or progression of age-related cataracts.

The results of our analysis tend to confirm the belief that nutrients provided as a component of food (Table 1) are superior to mono-nutrient type supplements (Table 2) for the prevention and delay of cataract disease. It appears a good diet is still central to vision-related health, however, small-moderate doses of some antioxidants may still be beneficial in reducing the progression and incidence of specific types of cataract. In general, the literature suggests diets high in fruit and vegetables, Vitamin C, zeaxanthin, lutein and multivitamin-mineral supplements are associated with lower disease rates, while supplemental forms of selenium and Vitamin E had little effect. 

Indicators and biomarkers of a healthy diet such as the healthy diet index, plasma antioxidant status and fruit and vegetable intake (Table 1) are associated with slower disease progression in those with moderate disease status, suggesting early intervention with a good diet is warranted [81]. A high intake of fruit and vegetables reduced the incidence of cataracts by 62% when data was appropriately adjusted, suggesting the simple intervention of consuming the recommended daily intake of fruit and vegetables is valid in this population group [74]. 

Lutein and zeaxanthin are the most abundant carotenoid in lens and have been shown to reduce damage from reactive species in cultured lens epithelial cells [80]. High dietary intake of lutein and zeaxanthin was associated with a 23% lower incidence of nuclear cataracts [80]. Women and men with carotenoid (lutein/zeaxanthin) intakes of approximately 4 to 6 mg/day have reduced rates of cataract extraction [69], demonstrating that dietary intake can not only reduce the incidence but delay disease progression.

Dietary (Table 1) or low dose supplemental (Table 2) Vitamin C reduces the incidence and progression of disease, demonstrated in the majority of the 9 studies. However, a study comparing Vitamin C as part of a multivitamin-mineral (60 mg/day) versus a Vitamin C supplement (1000 mg/day) showed the high dose, single nutrient actually increased the risk of cataracts, while there was no change in the multivitamin-mineral group [15]. Also, Zheng-Selin (2013) [83] reported that a high dose of Vitamin C increased the risk of cataract disease by 21% and Vitamin E increased the risk by 57%. Studies investigating the plasma concentrations of ascorbic acid support findings from the dietary supplement studies that reported higher ascorbic acid concentrations were associated with a lower incidence of cataracts. A small-to-moderate dose of Vitamin C (less than 100 mg/day) appears optimal, while we caution the recommendation of high dose supplemental Vitamin C, deemed to be equal or greater than 500 mg/day, particularly when taken chronically. Intakes of 250 mg/day of Vitamin C did reduce the signs and symptoms of cataracts in a three-month intervention trial [84], suggesting a short-term dose of Vitamin C may benefit those already diagnosed. The value long-term is still in question. 

While food contains a complex array of phytochemicals, nutrients and satiety factors proving a range of health benefits, ensuring adequate concentrations of the active ingredients reach the ocular region is a challenge. The intake of a multivitamin and mineral supplement also reduced the incidence of cataract disease, with Centrum^TM^ being the most commonly used brand. Centrum^TM^ contains a large range of nutrients in amounts reflecting a small percentage of the recommended daily allowance, as such is a true dietary supplement rather than an ergogenic aide. One capsule daily reduced the incidence of cataracts by 3% when taken for less than 10 years and by 6% when taken longer, suggesting the benefit for multivitamin intake is small, but useful long-term [83].

Mares-Perlman [85], reported a protective effect of supplementary Vitamin E (Table 2) reducing the incidence of cataracts by 10%, while dietary Vitamin E reduced disease incidence by 80%, confirming the theory that dietary intake is generally more effective than supplements. 

In summary, while the literature is mixed, there appears to be a general consensus that a diet high in fruit and vegetables containing Vitamin C, E, A and multivitamin-mineral supplements may be protective against cataracts. While the majority of dietary intake studies show a reduction in the risk of disease and disease progression, the strongest reductions were obtained with diets high in vitamin C, E and A, with lesser effects for diets rich in carotenoids and selenium. Data on supplemental antioxidants provided as low dose multivitamin appear generally positive, while results obtained using single nutrient antioxidants ranged from moderately effective to possibly harmful. Based on current data, a healthy diet and a multivitamin supplement may offer protection against cataracts. If this is the case, it is important to consider how these nutrients are able to reach the lens in sufficient quantities to be effective in protecting the lens from oxidative damage. In order to understand this process, a more thorough understanding of lens structure and function is required so that strategies to enhance the delivery and accumulation of these nutrients particularly in the lens nucleus can be identified, and harnessed to restore depleted antioxidant levels with advancing age. 

## 4. Lens Structure and Function

**Figure 3:**
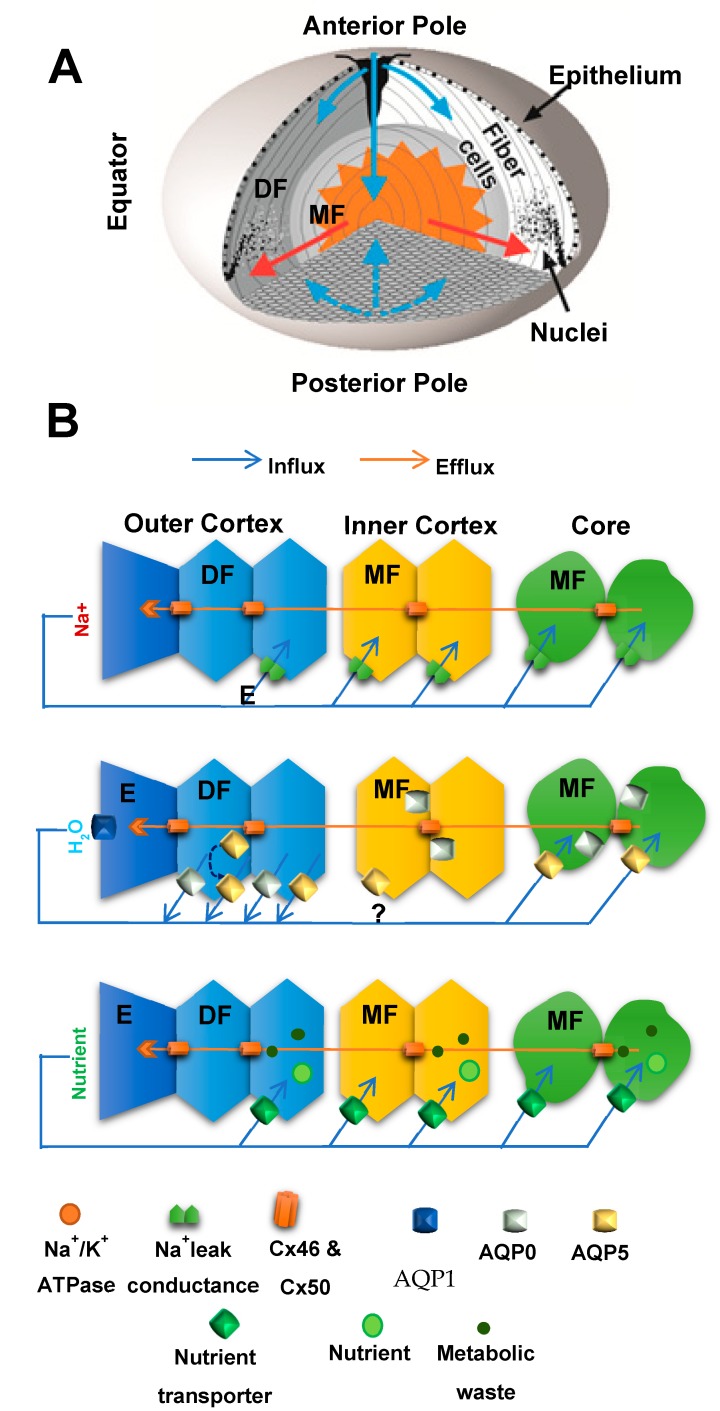
Lens structure and function (**A**) 3-D representation of the microcirculation model, showing ions and fluid fluxes that enter the lens at both poles via the extracellular space (blue arrows) before crossing fiber cell membranes and exiting the lens at the equator via an intracellular pathway (red arrows) mediated by gap junctions. (**B**) Equatorial cross-sections showing how the spatial differences in the distribution of ion channels and transporters between the epithelium (E), differentiating (DF) and mature (MF) fiber cells that generate the circulating flux of Na^+^ ions (*top*) that drives isotonic fluid fluxes (*middle*) which in turn deliver nutrients to and remove metabolic waste from the MF cells (*bottom*).

The transparency of the lens is closely linked to the unique structure and function of its fiber cells. These highly differentiated cells are derived from equatorial epithelial cells, which exit the cell cycle and embark upon a differentiation process that produces extensive cellular elongation, the loss of cellular organelles such as mitochondria and nuclei, and the expression of fiber-specific proteins [93,94]. Fiber cells elongate until fibers from opposite hemispheres meet at the poles and interdigitate to form the lens sutures [95]. Since this process continues throughout life, a gradient of fiber cells at different stages of differentiation is established around an internalized core (nucleus) of mature, anucleate fiber cells that were laid down in the embryo (Figure 3A). While the transparent properties of the lens are a direct result of its highly ordered tissue architecture, the lens is not a purely passive optical element. Maintenance of its architecture requires special mechanisms not only to supply the deeper lying fiber cells with nutrients, but also to control the volume of these cells. Because of its size, the avascular lens cannot rely on passive diffusion alone to transport nutrients and antioxidants from the surrounding humours to deeper lying cells, or to transport waste products back to the surface. Instead, it has been proposed that the lens operates an internal microcirculation system, which maintains lens transparency by delivering nutrients to the lens core faster than would occur by passive diffusion alone [96,97,98].

A common feature of all vertebrate lenses studied to date is the existence of a standing flow of ionic current that is directed inward at the poles and outward at the equator (Figure 3A). Mathias et al [98] have proposed that these currents measured at the lens surface represent the external portion of a circulating ionic current that drives a unique internal microcirculatory system that maintains fiber cell homeostasis and therefore lens transparency. Briefly, this model of lens transport states that a circulating current of Na^+^ ions, primarily enters at the poles and travels into the lens via the extracellular clefts between fiber cells (Figure 3B). Na^+^ crosses fiber cell membranes, and returns towards the surface via an intercellular pathway mediated by gap junction channels, where it is actively removed by Na^+^ pumps concentrated at the lens equator (Figure 3B, *top panel*). This circulating ionic current creates a net flux of ions that in turn generates fluid flow (Figure 3B, *middle panel*). The accompanying extracellular flow of water convects nutrients towards the deeper lying fiber cells, while the outward intercellular flow, driven by a hydrostatic pressure gradient [99], removes wastes and creates a well-stirred intracellular compartment (Figure 3B, *bottom panel*). Thus active pumping of Na^+^ by surface cells is able to regulate the ion composition of inner fiber cells and allows them to maintain the ion gradients necessary to power a variety of secondary active transporters that mediate steady state volume regulation and nutrient/antioxidant uptake [97].

While the evidence in favour of the circulation system has been accumulating over many years, it is not universally accepted [50,100]. The main criticisms have centred around our inability, to date, to actually visualize ion and fluid fluxes within the lens [100] and the perceived need for active metabolism in the lens nucleus. In an effort to measure water fluxes, Candia et al [101], employed Ussing chambers to measure regional water fluxes and showed that water influx at the poles is equal to water efflux at the equator, data which supports the existence of a circulating water fluxes through the lens. Utilizing a novel combination of MRI and confocal microscopy, Vaghefi et al [102,103] have visualized for the first time, the ion and fluid fluxes within the lens that were originally predicted by the microcirculation system. Furthermore, to visualize the delivery of solutes by the microcirculation system Vaghefi et al., have developed MRI imaging protocols that can visualize the rate of penetration of a variety of extracellular contrast agents of varying molecular size into the different regions of the lens [104]. Utilizing this approach, they have shown solute delivery to the lens core is not only dependent on the size of the extracellular tracer molecule, and also occurs at a rate faster than predicted for passive diffusion alone, but also that solute delivery is abolished if the microcirculation system is inhibited [104]. Thus by analogy, it would appear that the delivery of nutrients and antioxidants to the core of the lens is an active process that occurs via an extracellular route which is driven by the lens microcirculation system. 

To complete the delivery of nutrients and antioxidants to the core of the lens, the mature cells in this region of the lens need to be able to accumulate the solutes convected to them via the microcirculation system. In this regard Lim et al., have shown that mature fiber cells in the lens core differentially express a wide range of Na^+^-dependent and independent transporters that are capable of nutrient and antioxidant uptake in this region of the lens [105,106,107,108,109,110]. Furthermore, it is emerging that distinct metabolic compartments exist in the lens [111], which is not surprising considering that the peripheral differentiating fiber cells contain mitochondria and can perform aerobic metabolism, but mature fiber cells are devoid of mitochondria and rely on anaerobic metabolism to provide their metabolic requirements. This implies that in the different regions of the lens distinct metabolic pathways are utilized to maintain the levels of antioxidants that protect against cataract formation. It follows therefore, that the dysfunction of those region specific pathways is the underlying cause of the different etiologies of cortical and nuclear cataract, which in turn explains the variable efficacy of the different exogenous antioxidant supplements on the two types of cataract observed in epidemiological studies. 

## 5. Conclusions and Future Strategies

With a globally aging population, age related cataracts has grown to epidemic proportions, placing severe pressures on global and local health systems. Since the initiation of lens cataract is strongly associated with oxidative damage, the use of exogenous antioxidant interventions has been advocated as a strategy to slow the progression of lens cataract. However, the majority of these studies are ineffective in slowing down cataract progression. Rather, it appears that a combination and/or a range of endogenous nutrients may be more effective in slowing the progression of the different forms of lens cataract. In this regard, our current understanding of lens structure and function needs to be incorporated into the design of more effective anti-cataract therapies. By designing nutritional strategies that take into account the underlying physiology of how specific nutrients and antioxidants are delivered, taken up, and metabolized to maintain and restore antioxidant levels in the different regions of the lens, appeals as a strategy to prevent both cortical and nuclear cataract, and thereby avert the looming cataract epidemic facing our aging population.

## Figures and Tables

**Table 1:** Studies investigating antioxidant-rich food on disease progression.

**Vitamin C**				
**Author**	**Any CAT**	**NUC**	**CX**	**PSC**
Christen, 2008 [71]	RR 1.00; 95% CI 0.86–1.16; *P* = 0.61	-	-	-
Dherani, 2008 [72]	OR 0.64; 95% CI 0.48–0.85; *P* < 0.01	OR 0.62; 95% CI 0.4–0.96; *P* = 0.06	OR 0.62; 95% CI 0.40–0.97; *P* = 0.10	OR 0.59; 95% CI 0.35–0.99; *P* = 0.10
Jaques, 2001 [73]	-	OR 0.31; 95% CI 0.16–0.58; *P* = 0.003	-	-
Pastor-Valero, 2013 [74]	OR 0.46; 95% CI 0.24–0.88; *P* = 0.047	-	-	-
Ravindran, 2011 [75]	Plasma levels: OR 0.61; 95% CI 0.57–0.82; *P* < 0.0001Dietary OR 0.78; 95% CI 0.62–0.98; *P* = 0.006	OR 0.66; 95% CI 0.54–0.80; *P* < 0.0001	OR 0.70; 95% CI 0.54–0.90; *P* < 0.002	OR 0.58; 95% CI 0.45–0.74; *P* < 0.00003
Tan, 2008 [76]	-	OR 0.55; 95% CI 0.36–0.86; *P* = 0.045	OR 0.94; 95% CI 0.63–1.40; *P* = 0.698	OR 1.15; 95% CI 0.06–2.23; *P* = 0.954
Theodoropoulou, 2014 [77]	OR 0.50; 95% CI 0.39–0.64; *P* < 0.001	OR 0.55; 95% CI 0.41–0.72; *P* < 0.001	OR 0.62; 95% CI 0.37–1.04; *P* = 0.071	OR 0.30; 95% CI 0.19–0.49; P<0.001
Valero, 2002 [12]	OR 0.70; 95% CI 0.44–1.13; *P* = 0.04	OR 0.56; 95% CI 0.38–0.82	OR 0.92; 95% CI 0.60–1.40	OR 0.75; 95% CI 0.51–1.05
Yoshida, 2007 [78]	Men: OR 0.65; 95% CI 0.42–0.97; *P* = 0.094 Women: OR 0.59; 95% CI 0.43–0.89; *P* = 0.047	-	-	-
**Vitamin E**				
**Author**	**Any CAT**	**NUC**	**CX**	**PSC**
Christen, 2008 [71]	RR 0.92; 95% CI 0.80–1.06; *P* = 0.39	-	-	-
Jaques, 2001 [73]	-	OR 0.45; 95% CI 0.23–0.86; *P* = 0.06	-	-
Pastor-Valero, 2013 [74]	OR 0.46; 95% CI 0.24–0.88; *P* = 0.944	-	-	-
Tan, 2008 [76]	-	OR 0.73; 95% CI 0.47–1.13; *P* = 0.155	OR 0.91; 95% CI 0.62–1.33; *P* = 0.944	OR 0.95; 95% CI 0.50-1.83; P=0.597
Theodoropoulou, 2014 [77]	OR 0.50; 95% CI 0.38–0.66; *P* < 0.001	OR 0.50; 95% CI 0.36–0.69; *P* < 0.001	OR 0.71; 95% CI 0.41–1.25; *P* = 0.238	OR 0.42; 95% CI 0.26–0.68; *P* < 0.001
Valero, 2002 [12]	OR 0.77; 95% CI 0.48–1.24; *P* = 0.60	OR 0.81; 95% CI 0.50–1.28	OR 1.00; 95% CI 0.59–1.72	OR 1.16; 95% CI 0.71–1.90
**Vitamin A (Retinol)**				
**Author**	**Any CAT**	**NUC**	**CX**	**PSC**
Dherani, 2008 [72]	OR 0.58; 95% CI 0.37–0.91; *P* < 0.02	OR 0.56; 95% CI 0.33–0.46; *P* = 0.04	OR 0.69; 95% CI 0.38–1.26; *P* = 0.20	OR 0.69; 95% CI 0.39–1.23; *P* = 0.20
Tan, 2008 [76]	-	OR 066; 95% CI 0.42–10.3; *P* = 0.056	OR 0.84; 95% CI 0.56–1.25; *P* = 0.305	OR 1.04; 95% CI 0.54–2.02; *P* = 0.604
Theodoropoulou, 2014 [77]	OR 1.47; 95% CI 1.150–1.88; *P* = 0.002	OR 1.46; 95% CI 1.11–1.92; *P* = 0.007	OR 1.02; 95% CI 0.51–2.02; *P* = 0.962	OR 1.88; 95% CI 1.35–2.63; *P* < 0.001
Valero, 2002 [12]	OR 0.82; 95% CI 0.50–1.03; *P* = 0.21	Plasma levels: OR 1.67; 95% CI 1.02–2.72	Plasma levels: OR 1.82; 95% CI 1.09–3.08	Plasma levels: OR 1.22; 95% CI 0.73–2.03
**Selenium**				
**Author**	**Any CAT**	**NUC**	**CX**	**PSC**
Valero, 2002 [12]	OR 0.97; 95% CI 0.60–1.58; *P* = 0.34	OR 0.71; 95% CI 0.48–1.04	OR 0.88; 95% CI 0.58–2.46	OR 1.03; 95% CI 0.70–1.51
**Carotenoids**				
**Author**	**Any CAT**	**NUC**	**CX**	**PSC**
Christen, 2008 [71]	Lutein/zeaxanthin: RR 0.82; 95% CI 0.71–0.95; *P* = 0.045 Alpha-carotene: RR 0.96; 95% CI 0.84–1.11; *P* = 0.77 Beta-carotene: RR 0.89; 95% CI 0.77–1.02; *P* = 0.27 Beta-cryptoxanthin: RR 0.92; 95% CI 0.80–1.06; *P* = 0.19 Lycopene: RR 0.96; 95% CI 0.52–30.7; *P* = 0.6	-	-	-
Delcourt, 2006 [79]	Plasma levels:Lutein: OR 0.82; 95% CI 0.48–1.41; *P* = 0.48 Zeaxanthin: OR 0.57; 95% CI 0.34–0.95; *P* = 0.03 Beta-carotene: OR 0.69; 95% CI 0.40–1.19; *P* = 0.17 Alpha-carotene: OR 0.69; 95% CI 0.4–1.19; *P* = 0.17 Beta-cryptoxanthin: OR 0.71; 95% CI 0.42–1.20; *P* = 0.20 Lycopene: OR 1.17; 95% CI 0.68–2.01; *P* = 0.58	Plasma levels:Lutein: OR 0.60; 95% CI 0.24–1.47; *P* = 0.26 Zeaxanthin: OR 0.25; 95% CI 0.08–0.71; *P* = 0.004 Beta-carotene: OR 0.42; 95% CI 0.16–1.12; *P* = 0.07 Alpha-carotene: OR 0.76; 95% CI 0.29–2.03; *P* = 0.52 Beat-cryptoxanthin: OR 0.70; 95% CI 0.29–1.68; *P* = 0.40 Lycopene: OR 1.01; 95% CI 0.41–2.51; *P* = 0.99	Plasma levels:Lutein: OR 0.75; 95% CI 0.23–2.47; *P* = 0.63 Zeaxanthin: OR 1.09; 95% CI 0.37–3.26; *P* = 0.83 Beta-carotene: OR 1.10; 95% CI 0.28–4.26; *P* = 0.93 Alpha-carotene: OR 0.97; 95% CI 0.32–2.91; *P* = 0.96 Beta-cryptoxanthin: OR 1.49; 95% CI 0.48–4.68; *P* = 0.49 Lycopene: OR 0.92; 95% CI 0.27–3.13; *P* = 0.59	Plasma levels:Lutein: OR 1.26; 95% CI 0.52–3.07; *P* = 0.60 Zeaxanthin: OR 0.84; 95% CI 0.34–2.07; *P* = 0.68 Beta-carotene: OR 0.51; 95% CI 0.19–1.36; *P* = 0.16 Alpha-carotene: OR 0.72; 95% CI 0.30–1.73; *P* = 0.46 Beta-cryptoxanthin: OR 0.42; 95% CI 0.15–1.18; *P* = 0.09 Lycopene: OR 1.19; 95% CI 0.49–2.88; *P* = 0.70
Dherani, 2008 [72]	Lutein: OR 0.66; 95% CI 0.43–1.02; *P* = 0.06 Zeaxanthin: OR 0.66; 95% CI 0.45–0.96; *P* < 0.03 Beta-cryptoxanthin: OR 0.88; 95% CI 0.63–1.23; *P* = 0.50 Alpha-carotene: OR 0.69; 95% CI 0.50–0.95; *P* < 0.05 Beta-carotene: OR 0.77; 95% CI 0.45–1.32; *P* = 0.30 Lycopene: OR 0.78; 95% CI 0.49–1.23; *P* = 0.20 Alpha-tocopherol: OR 0.58; 95% CI 0.36–0.94; *P* = 0.04 Gamma-tocopherol: OR 0.75; 95% CI 0.57–0.98; *P* = 0.06	Lutein: OR 0.75; 95% CI 0.44–1.31; *P* = 0.30 Zeaxanthin: OR 0.71; 95% CI 0.43–1.17; *P* = 0.20 Beta-cryptoxanthin: OR 0.83; 95% CI 0.56–1.22; *P* = 0.30 Alpha-carotene: OR 0.74; 95% CI 0.48–1.14; *P* = 0.20 Beta-carotene: OR 0.75; 95% CI 0.44–1.26; *P* = 0.20 Lycopene: OR 0.83; 95% CI 0.50–1.37; *P* = 0.40 Alpha-tocopherol: OR 0.60; 95% CI 0.30–1.20; *P* = 0.10 Gamma-tocopherol: OR 0.99; 95% CI 0.62–1.58; *P* = 0.90	Lutein: OR 0.53; 95% CI0.28–1.02; *P* = 0.10 Zeaxanthin: OR 0.58; 95% CI 0.30–1.12; *P* = 0.10 Beta-cryptoxanthin: OR 0.93; 95% CI 0.54–1.56; *P* = 0.90 Alpha-carotene: OR 0.86; 95% CI 0.60–1.22; *P* = 0.70 Beta-carotene: OR 1.02; 95% CI 0.52–1.99; *P* = 0.90 Lycopene: OR 1.17; 95% CI 0.54–2.53; *P* = 0.70 Alpha-tocopherol: OR 0.58; 95% CI 0.25–1.35; *P* = 0.20 Gamma-tocopherol: OR 0.84; 95% CI 0.44–1.00; *P* = 0.50	Lutein: OR 0.72; 95% CI 0.30–1.71; *P* = 0.40 Zeaxanthin: OR 0.84; 95% CI 0.43–1.67; *P* = 0.60 Beta-cryptoxanthin: OR 1.06; 95% CI 0.54–2.05; *P* = 0.80 Alpha-carotene: OR 0.80; 95% CI 0.39–1.65; *P* = 0.60 Beta-carotene: OR 0.71; 95% CI 0.30–1.68; *P* = 0.40 Lycopene: OR 0.68; 95% CI 0.29–1.61; *P* = 0.30 Alpha-tocopherol: OR 1.11; 95% CI 0.42–2.91; *P* = 0.90 Gamma-tocopherol: OR 1.16; 95% CI 0.69–1.93; *P* = 0.60
Jaques, 2001 [73]	-	Alpha-carotene: OR 0.71; 95% CI 0.37–1.35; *P* = 0.39 Beta-carotene: OR 0.52; 95% CI 0.28–0.97; *P* = 0.08 Beta-cryptoxanthin: OR 0.68; 95% CI 0.34–1.35; *P* = 0.06 Lutein/zeaxanthin: OR 0.52; 95% CI 0.29–0.91; *P* = 0.08 Lycopene: OR 1.16; 95% CI 0.63–2.16; *P* = 0.79	-	-
Moeller, 2008 [80]	-	Lutein: OR 0.68; 95% CI 0.48–0.97; *P* = 0.04 Zeaxanthin: OR 0.68; 95% CI 0.47–0.98; *P* = 0.01	-	-
Tan, 2008 [76]	-	Beta-carotene: OR 1.09; 95% CI 0.69–1.72; *P* = 0.715	Beta-carotene: OR 1.06; 95% CI 0.7–1.6; *P* = 0.854	Beta-carotene: OR 0.76; 95% CI 0.37–1.59; *P* = 0.317
Theodoropoulou, 2014 [77]	Carotene: OR 0.56; 95% CI 0.45–0.69; *P* < 0.001	Carotene: OR 0.50; 95% CI 0.39–0.65; *P* < 0.001	Carotene: OR 0.68; 95% CI 0.43–1.05; *P* = 0.084	Carotene: OR 0.58; 95% CI 0.40–0.86; *P* = 0.007
Valero, 2002 [12]	Beta-carotene: OR 0.82; 95% CI 0.51–1.33; *P* = 0.34 Alpha-carotene: OR 0.64; 95% CI 0.39–1.04; *P* = 0.07 Beta-cryptoxanthin: OR 0.97; 95% CI 0.61–1.56; *P* = 0.41 Lutein: OR 1.00; 95% CI 0.64–1.64; *P* = 0.78 Zeaxanthin: OR 0.99; 95% CI 0.61–1.60; *P* = 0.83 Lycopene: OR 1.11; 95% CI 0.69–1.78; *P* = 0.81	Blood lycopene: OR 1.55; 95% CI 1.00–2.38	Blood lycopene: OR 1.20; 95% CI 0.76–1.90	Blood lycopene: OR 1.34; 95% CI 0.87–2.07
**Other**				
**Author**	**Any CAT**	**NUC**	**CX**	**PSC**
Mares, 2010 [81]	-	High vs low HEI score: OR 0.63; 95% CI 0.43–0.91	-	-
Rautiainen, 2014 [82]	Highest vs lowest TAC quintile: OR 0.87 95% CI 0.79–0.96; *P* = 0.03	-	-	-

**Table 2:** Studies investigating antioxidant rich supplements on cataract incidence.

Author	Sample Size (*n*), Age (years)	Nutrients Examined	Key Findings
Multi-Vitamins			
Mares-Perlman, 2000 [85]	*n* = 3089	Supplementary Multivitamin, Vitamin C, Vitamin E	The 5-year risk for any CAT was 60% lower for multivitamins or any supplement use containing Vitamin C or E for more than 10 years. 10-year multivitamin use lowered the risk for NUC and CX but not for PSC (OR 0.6, 95% CI 0.4–0.9; OR 0.4, 95% CI 0.2–0.8; and OR, 0.9 95% CI 0.5–1.9; respectively).
Kuzniarz, 2001 [11]	*n* = 2873, 49–97 years	Supplementary Vitamin A, Thiamine, Riboflavin, Niacin, Pyridoxine, Folate, Vitamin B12	Use of multivitamin supplements was associated with reduced prevalence of NUC, OR 0.6, 95% CI 0.4–1.0, *P* =0.05. For both NUC and CX, longer duration of multivitamin use was associated with reduced prevalence (NUC, trend *P* = 0.02; CX, trend *P* = 0.03). Use of thiamin supplements was associated with reduced prevalence of NUC (OR 0.6, 95% CI 0.4–1.0, *P* = 0.03, dose trend *P* = 0.03) and CX (OR 0.7, 95% CI 0.5–0.9, *P* = 0.01, dose trend *P* = 0.02). Riboflavin (OR 0.8, 95% CI 0.6–1.0, *P* = 0.05) and niacin (OR 0.7, 95% CI 0.6–1.0, *P* = 0.04) supplements exerted a weaker protective influence on CX. Vitamin A supplements were protective against NUC (OR 0.4, 95% CI 0.2–0.8, *P* = 0.01, dose trend *P* = 0.01). Folate (OR 0.4, 95% CI 0.2–0.9, *P* = 0.03) appeared protective for NUC, whereas both folate (NUC 0.6, 95% CI 0.3–0.9, *P* = 0.01, dose trend *P* = 0.04) and Vitamin B12 supplements (OR 0.7, 95% CI 0.5–1.0, *P* = 0.03, dose trend *P* = 0.02) were strongly protective against CX.
Age-Related Eye Disease Study Research Group, 2006 [86]	*n* = 4590, -	Supplementary Centrum^TM^ Multivitamin (Vitamin A, E, C, B1, B2, B12, B6, D, Folic acid, Niacinamide, Biotin, Pantothenic acid, Calcium, Phosphorus, Iodine, Iron, Magnesium, Copper Zinc)	Centrum^TM^ use is associated with a reduction in any lens opacity progression (OR 0.84, 95% CI 0.72–0.98, *P =* 0.025). Also protective for nuclear opacity events (OR 0.75, 95% CI 0.61–0.91, *P =* 0.004).
Zheng Selin, 2013 [83]	*n* = 31120, 45–79 years	Supplemental Vitamin C, Vitamin E, Low dose multivitamins	The multivariable- adjusted HR for Vitamin C supplements only was 1.21 (95% CI 1.04–1.41) in compared to non-users. The HR for long-term Vitamin C users (≥10 years before baseline) was 1.36 (95% CI 1.02–1.81). The HR for Vitamin E use only was 1.59 (95% CI 1.12–2.26). Use of multivitamins only or multiple supplements in addition to Vitamin C or E was not associated with cataract risk.
Single Vitamins			
Christen, 2004 [87]	*n* = 39876, ≥ 45 years	Supplementary Beta-carotene (50mg.d^-1^, alternate days)	129 CAT in the beta-carotene group and 133 in the placebo group (RR 0.95, 95% CI 0.75–1.21). For cataract extraction, there were 94 cases in the beta-carotene group and 89 cases in the placebo group (RR 1.04, 95% CI 0.78–1.39).
Christen, 2008 [88]	*n* = 39876, ≥ 45 years	Supplementary Vitamin E (600 IU.d^-1^, alternate days)	No significant difference between the Vitamin E and placebo groups in the incidence of CAT (RR 0.96; 95% CI 0.88–1.04). No significant effects of Vitamin E on the incidence of NUC (RR 0.94; 95% CI 0.87–1.02), CX (RR 0.93; 95% CI 0.81–1.06), or PSC (RR 1.00; 95% CI 0.86–1.16).
Christen, 2015 [89]	*n* = 35533, ≥50 years	Supplementary Selenium (200 μg.d^-1^ from L-selenomethionine), Vitamin E (400 IU.d^-1^ of all rac-α-tocopheryl acetate)	185 CAT in the selenium group and 204 in placebo (HR 0.91; 95 % CI 0.75–1.11; *P* = 0.37). For Vitamin E, there were 197 cases in the Vitamin E group and 192 in placebo (HR 1.02; 95 % CI 0.84–1.25; *P* = 0.81)
Christen,2010 [90]	*n* = 11545, ≥50 years	Supplementary Vitamin E (400 IU.d^-1^, alternate days) Vitamin C (500 mg.d^-1^ alternate days)	579 CAT in the Vitamin E treated group and 595 in the Vitamin E placebo group (HR 0.99; 95% CI 0.88–1.11). For Vitamin C, there were 593 cataracts in the treated group and 581 in the placebo group (HR 1.02; 95% CI 0.91–1.14).
Christen,2002 [91]	*n* = 22071	Supplementary Beta-carotene (50 mg.d^-1^, alternate days)	No difference between the beta-carotene and placebo groups in the overall incidence of CAT (998 cases vs 1017 cases; RR 1.00; 95% CI 0.91–1.09) or CAT extraction (584 vs 593; RR 1.00; 95% CI 0.89–1.12).
Ferringo, 2005 [92]	*n* = 1020	Supplementary Vitamin A, Vitamin C, Vitamin E, Beta-carotene	High Vitamin C levels were associated with a protective effect on NUC (OR 0.54; 95% CI 0.30, 0.97) and PSC (OR: 0.37; 95% CI: 0.15–0.93). High Vitamin E levels were associated with increased prevalence of CX (OR 1.99; 95% CI 1.02–3.90), PSC (OR 3.27; 95% CI 1.34–7.96) and of any CAT (OR 1.86; 95% CI 1.08–3.18).
Rautiainen, 2009 [15]	*n* = 24593, 49–83 years	Supplementary Vitamin C	HR of Vitamin C supplement users compared with that for nonusers was 1.25 (95% CI 1.05, 1.50). The HR for the duration of 10 y of use before baseline was 1.46 (95% CI 0.93, 2.31). The HR for the use of multivitamins containing Vitamin C was 1.09 (95% CI 0.94, 1.25). Among women aged 65 y, Vitamin C supplement use increased the risk of CAT by 38% (95% CI 12%, 69%).
The REACT Group,2002 [84]	*n* = 297	Supplementary Vitamin E (200 mg all-rac alpha-tocopherol acetate), Vitamin C (250 mg ascorbic acid), and b-carotene (6 mg)	After two years of treatment, there was a small positive treatment effect in U.S. patients (*p* = 0.0001); after three years a positive effect (*p* = 0.048) in both the U.S. and the U.K. groups. The positive effect in the U.S. group was even greater after three years: (IPO = 0.389 (Vitamin) vs. IPO = 2.517 (placebo); *p* = 0.0001).
